# Quantitative RNA-Seq analysis in non-model species: assessing transcriptome assemblies as a scaffold and the utility of evolutionary divergent genomic reference species

**DOI:** 10.1186/1471-2164-13-361

**Published:** 2012-08-01

**Authors:** Emily A Hornett, Christopher W Wheat

**Affiliations:** 1 Department of Biological Sciences, University of Helsinki, PL 65, Viikinkaari 1, 00014, Helsinki, Finland

## Abstract

**Background:**

How well does RNA-Seq data perform for quantitative whole gene expression analysis in the absence of a genome? This is one unanswered question facing the rapidly growing number of researchers studying non-model species. Using *Homo sapiens* data and resources, we compared the direct mapping of sequencing reads to predicted genes from the genome with mapping to *de novo* transcriptomes assembled from RNA-Seq data. Gene coverage and expression analysis was further investigated in the non-model context by using increasingly divergent genomic reference species to group assembled contigs by unique genes.

**Results:**

Eight transcriptome sets, composed of varying amounts of Illumina and 454 data, were assembled and assessed. Hybrid 454/Illumina assemblies had the highest transcriptome and individual gene coverage. Quantitative whole gene expression levels were highly similar between using a *de novo* hybrid assembly and the predicted genes as a scaffold, although mapping to the *de novo* transcriptome assembly provided data on fewer genes. Using non-target species as reference scaffolds does result in some loss of sequence and expression data, and bias and error increase with evolutionary distance. However, within a 100 million year window these effect sizes are relatively small.

**Conclusions:**

Predicted gene sets from sequenced genomes of related species can provide a powerful method for grouping RNA-Seq reads and annotating contigs. Gene expression results can be produced that are similar to results obtained using gene models derived from a high quality genome, though biased towards conserved genes. Our results demonstrate the power and limitations of conducting RNA-Seq in non-model species.

## Background

Massively parallel sequencing of RNA, known as RNA-Seq, provides unprecedented access to sequence and expression variation in the transcriptome [1,2] and allows for additional insights into alternative splicing [3], cis vs. trans gene regulation [4], and micro-RNA dynamics [5]. RNA-Seq experiments for gene expression analysis typically involve mapping 10’s of millions of short sequencing reads onto the reference dataset (scaffold) of a model species, whose genome has been sequenced and gene models determined [6]. As next generation sequencing becomes more affordable (see [7] for an insightful discussion of hidden costs), RNA-Seq is becoming increasingly attractive for quantitative studies of differential gene expression in non-model species, for which there is often much knowledge of the evolution and ecology but little or no genomic resources.

The non-model species community is rapidly harnessing the transcriptome, with an explosion of RNA-Seq studies published over the past 5 years, predominantly using the longer sequencing reads of the 454 FLX technology for the generation of EST databases containing sequence and SNP information [8-12]. This community is now also beginning to use the Illumina platform with excellent results, further decreasing the cost for transcriptome database construction and SNP identification [13-16]. However, although the Illumina sequencing platform is the main workhorse for quantitative transcript analysis, only a handful of studies have begun to use this platform to study expression variation in non-model species (e.g. [17,18]), primarily due to concerns over read mapping accuracy in the absence of a genome scaffold (e.g. [9]). Given that the number of RNA-Seq studies in non-model species is expected to rapidly increase [19] there is a pressing need to assess the performance of RNA-Seq in the non-model species context.

A researcher wishing to conduct RNA-Seq in a species lacking genomic resources faces a series of currently unanswered questions. These initially are queries of how much data is necessary to produce informative significant results, the price of producing such data and what sequencing platform to use. There are also concerns over the quality of *de novo* transcriptomes and their utility as scaffolds for mapping RNA-Seq reads compared to a high quality genome, or indeed any genome for the target species. An additional problem faced by the non-model community is the ability to draw functional information from the *de novo* assembly and expression results. Combining reads per gene and annotating assembly contigs can be achieved by using the genome or predicted gene set of a related species as proxy. However, the level of bias and error this introduces is for the most part unknown, as is the effect of evolutionary distance between the proxy reference and the study species. Here we directly address these questions in detail using RNA-Seq data and genomic resources available for *Homo sapiens*.

## Results

### *de novo transcriptome assembly and assessment*

RNA-Seq data were assembled in various combinations into eight different transcriptome assemblies (TAs) to assess the relative performance of different sequencing technologies and their utility in combination. To clarify, a ‘read’ is the short sequence output of the sequencing platform (e.g. Illumina with 35-100 bp reads). A ‘contig’ is a contiguous sequence formed from two or more reads that are found to overlap. Three of the TAs were assembled using three replicate paired-end Illumina runs of similar size (TA_Illpr1 (15.3 Mb), TA_Illpr2 (14.7 Mb), TA_Illpr3 (14.0 Mb)). A fourth TA combined all of the reads from these datasets into one assembly (TA_Illprs, 28.5 Mb). The fifth also used all of this RNA-Seq data and incorporated an additional Illumina RNA-Seq dataset in order to determine the effect of increasing the volume of data upon assembly performance (TA_All_Ill, 35.3 Mb). The sixth TA was created using only 454 RNA-Seq reads (TA_454, 46.1 Mb), while the seventh used the three pairs of Illumina data plus this 454 dataset (TA_Illprs&454, 68.0 Mb). TA_All (71.0 Mb) was assembled using all of aforementioned RNA-Seq data (see Methods and Additional file 1: Table S 1).

#### Standard metrics of assembly quality

**Figure 1:**
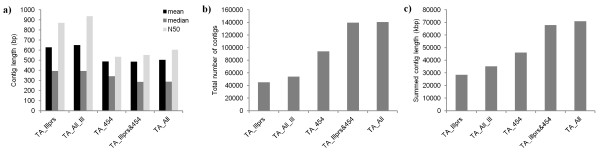
**Basic assembly metrics of five *de novo *transcriptome assemblies (TAs).** Comparison of the assembly metrics for five TAs generated from different data sources: **a)** the mean, median and N50 TA contig length, **b)** the total number of contigs in the TA, and **c)** the summed contig length.

We initially assessed the *de novo* TAs by comparing several standard metrics commonly used in ascertaining the quality of an assembly [10]: total number of contigs; longest contig length; mean and median contig length; N50 (the median contig size, length weighted); and the summed contig lengths (i.e. raw size of the TA). The amount and type of RNA-Seq data incorporated into the eight TAs and the basic assembly metrics are summarized in Additional file 1 Table S1. For the three TAs created using replicate Illumina RNA-Seq data (TA_Illpr1, TA_Illpr2, TA_Illpr3), each gave near identical performance to each other but were all consistently inferior to the other five TAs in terms of the basic assembly metrics. We subsequently focussed upon quality comparisons of the remaining five TAs: TA_Illprs, TA_All_Ill, TA_454, TA_Illprs&454, and TA_All (Figure 1).

An optimal assembly will have near full length contigs similar to that expected from the actual transcriptome of the target species. The basic metrics shown in Figure 1a & b suggested that TA performance was best with Illumina data alone. TA_Illprs and TA_All_Ill had the largest contig mean, median and N50 lengths, whilst also having the lowest number of contigs. These metrics indicate that the TA composed solely of 454 reads (TA_454) was of poorer quality than those composed purely of Illumina reads. The assemblies comprised of both Illumina and 454 sequencing data (TA_Illprs&454 and TA_All) also had a comparatively small mean, median and N50 contig length. These hybrid assemblies also had the greatest number of contigs, containing more than twice as many as the TAs solely composed of Illumina reads. However, the optimal assembly will also have a large summed contig length, and this metric provided a very different set of conclusions (Figure 1c). For summed contig length, the hybrid assemblies performed much better than the pure assemblies with summed contigs lengths of: TA_Illprs&454: 67.96 Mb and TA_All: 71.05 Mb, both of which being approximately twice as large as TA_All_Ill (35.30 Mb) and over twice the size of TA_Illprs (28.49 Mb)*.* Thus, despite having larger contigs, the *de novo* transcriptomes composed of pure Illumina reads were overall much smaller.

#### de novo *transcriptome assembly coverage metrics*

The contrasting insights provided by the basic metrics illustrate their limited utility. Metrics based upon contig lengths (e.g. mean, median, N50) do not provide quantitative insights into how much of the target species transcriptome is represented in the *de novo* TA. For transcriptome assembly in the context of generating a scaffold for RNA-Seq mapping, optimising the representation of the transcriptome is critical since the only RNA-Seq data that is analysed is that which can be aligned to a scaffold. Here we calculated several additional metrics to gauge the quality of a *de novo* transcriptome assembly by taking advantage of the genomic resources for *Homo sapiens*.

**Figure 2:**
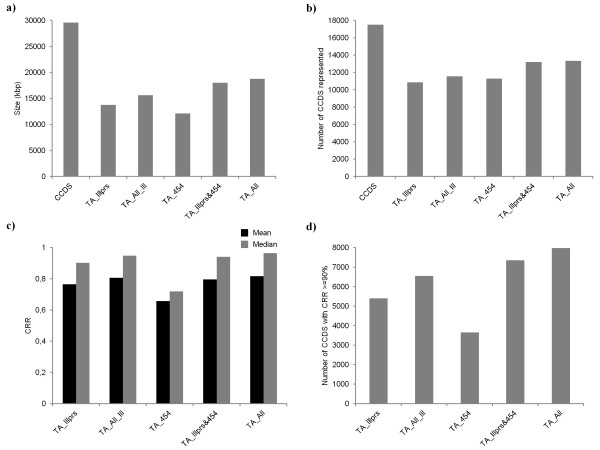
**Assessment of TA quality using genomic information. a)** the total size (kbp) of the TAs compared to the CCDS, adjusted so only the contig sequence that aligns to a CCDS is included; **b)** the total number of genes represented in each TA compared to the CCDS; **c)** the mean and median CRR (coverage of CCDS) in a TA; and **d)** the number of CCDS transcripts that have equal or greater than 90% CRR in the TA.

We assessed the integrity and completeness of the TAs in terms of their recapitulation of the *H. sapiens* predicted consensus coding sequence (CCDS) gene set. First, we quantified the size of the coding region of the *de novo* TAs in comparison to the summed length of all CCDS (Figure 2a). Given that one or more TA contigs may align to a given CCDS, we used BLASTn to identify these relationships and calculate the regions that were covered of each CCDS by at least one TA contig. That is, we only took the length of the CCDS that was covered regardless of the number of different TA contigs that covered those regions (i.e. if 5 TA contigs all aligned to nucleotides 150 to 550 of a given CCDS, the length covered was only 400 bp). Compared to the total size of the *H. sapiens* CCDS dataset, the hybrid assemblies TA_Illprs&454 and TA_All performed the best, covering 61% and 64% of the total size of the CCDS dataset respectively, while TA_454 had the smallest transcriptome coverage at 38%. Second, we counted the number of *H. sapiens* predicted genes (CCDS) hit by a TA contig in BLASTn searches (Figure 2b). The two hybrid assemblies had the greatest number of CCDS represented: 75% and 76% of the CCDS being hit by TA_Illprs&454 and TA_All respectively. Of note is that although TA_454 was the smallest in term of coding size (Figure 1a), its coverage of the transcriptome as estimated by the number of CCDS hit by this TA was similar to both of the TAs composed solely of Illumina reads (TA_454 64%; TA_Illprs 62%; TA_All_Ill 66%).

In determining how successful the coverage of the actual transcriptome was in the *de novo* TAs created here, we first note that the transcriptome from one tissue type is expected to contain fewer transcripts than that of the whole organism. One study estimates a figure of 70% of the genes of an organism’s genome [20]. Given that the CCDS dataset represents the entire transcriptome, and the RNA-Seq data obtained for this study derives from sequencing a human brain sample, we cannot therefore expect a coverage figure near 100%. In addition the RNA sample was not normalized, so the RNA-Seq data were likely to primarily contain highly transcribed genes and miss many lowly expressed transcripts. The largest TA, TA_All, is 18.8 Mb and hits 13,343 CCDS. This is 64% of the size of the CCDS dataset (29.6 Mb) and comprises 76% of the CCDS transcripts (n = 17,520).

While these two measures, total coding size and the number of genes represented in each TA, are very informative, knowing the proportion of each gene covered by TA contigs is also important (i.e. the completeness [21]). We therefore calculated what we term the contig reference ratio (CRR) for each of the CCDS to ascertain the actual coverage of the transcriptome for the different TAs. The CRR therefore is the ratio of the length of the CCDS uniquely covered by TA contigs divided by the length of that CCDS (e.g. a CRR of 0.9 means that 90% of that CCDS is covered by one or more TA contigs). This metric directly indicates the amount of sequence in the TA that is informative, as it is the coding regions that can be used to align to divergent species and identify synonymous and non-synonymous SNPs. We initially calculated the mean and median CRR across the five TAs to assess the average coverage per gene across the transcriptome (Figure 2c). The mean and median CRR values were fairly consistent between the different assemblies, with the exception of TA_454 that had lower coverage per CCDS and therefore would provide less sequence information per transcript. The remaining four TAs all had very high CRR median and mean values, showing that the majority of the CCDS represented in the TAs have very high coverage (Figure 2c).

Finally, a stringent assessment of assembly performance was performed by calculating the number of CCDS represented in a TA which had a >=90% CRR (Figure 2d). The Illumina only TAs, and especially the TA composed solely of 454 reads, performed poorly in comparison to the hybrid assemblies. The results of this metric are particularly informative when looked at in conjunction with the number of CCDS represented in each TA (Figure 2b). Although the number of CCDS hit by TA_454 was comparable to that of the two TAs composed purely of Illumina reads (TA_Illprs and TA_All_Ill), we can see that of those CCDS hit, the CRR is much lower. Indeed, of the 11,297 CCDS represented in TA_454, only 29.8% had a CRR of 0.9 or greater. This explains the discrepancy of the results for TA_454 of total size (Figure 2a) and number of CCDS represented (Figure 2b). TA_Illpairs had 50.8% of 10,859 CCDS represented with CRR > = 90% and TA_All_Ill has 58.0% of 11,565 CCDS represented with CRR > =90%. Using these ‘genomic’ quality metrics, we found that while the TA_All performed best, with 61.1% of the 13,343 CCDS represented having a CRR of > =90%, the other hybrid TA, TA_Illprs&454, was similar in performance with 56.4% of the 13,213 CCDS represented having a CRR of > =90%.

**Figure 3:**
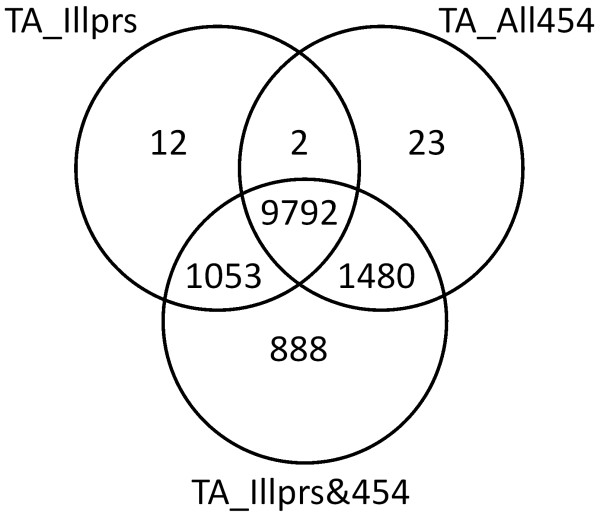
Venn diagram displaying the numbers of CCDS transcripts represented in each of three TAs.

**Figure 4:**
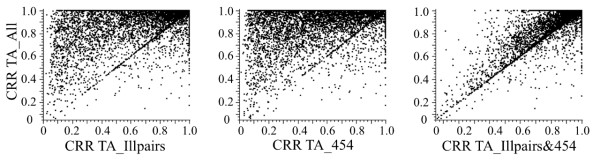
**Comparison of the coverage (CRR) of the *de novo *TAs.** The best quality transcriptome produced (TA_All) and three other TAs created using RNA-Seq from different sequencing methods were compared: panel of three graphs depicting the CRR of CCDS that are represented in all three TAs, each datapoint (black dots) represents the CRR of a CCDS.

Closer analysis of the number of genes represented in three of the TAs revealed that although there is a large area of overlap in the CCDS in common between TA_Illprs and TA_454 (n = 9794 CCDS), the TAs created from either Illumina or the 454 data hit different areas of the transcriptome (Figure 3). There were 1065 and 1503 CCDS unique to TA_Illprs and TA_454 respectively, suggesting that these different technologies sequenced different areas of given transcripts. This was assessed further by looking at the CRR values for each CCDS represented in the pure Illumina TA (TA_Illprs), the pure 454 TA (TA_454), and the largest hybrid TA (TA_All). The CRR of a particular CCDS was often much lower in the pure assemblies compared to the hybrid assembly (Figure 4). When the 454 and Illumina reads were combined in the other hybrid TA (TA_Illprs&454) the CRR of a given CCDS was much longer, indicating that these reads are providing complementary coverage rather than similar coverage of a given CCDS, with the results comparable in coverage to that of TA_All (Figure 4). This is similarly true when we look at the CRR of the single longest TA contig per CCDS (Additional file 2: Figure S 1); in the single platform TAs, although the Illumina data does generate longer TA contigs compared to the 454 data, their combined data results in TAs that have a greater number of longer contigs per CCDS. This derives from the much larger coverage of the transcriptome by Illumina runs coupled with the ability of the assembler we used to pull that data together correctly into contigs. Thus combining data from these two different sequencing platforms not only increased the number of CCDS, it also increased the coverage within a CCDS by bringing together contigs that hit different areas of that CCDS. In addition, the hybrid transcriptome assemblies resulted in contigs being formed for genes that were not present in assemblies composed of one type of sequencing platform (n = 888, Figure 3).

#### *Comparison of expression profiles produced using different* de novo *TA scaffolds*

In order to assess the performance of the different *de novo* TAs as scaffolds for RNA-Seq expression analysis, we compared the use of a TA as a mapping scaffold to that of a predicted gene set from the well characterized reference genome of *H. sapiens*. Since our focus in this study was upon the utility of *de novo* RNA-Seq for quantifying whole gene expression in non-model species, we assessed the accuracy and efficiency of drawing together sequencing reads that are from the same gene to calculate an expression value for that gene. In the latter part of this paper, we explore this mapping relationship in the non-model species case of using increasingly evolutionary divergent species (from the target species) for grouping contigs by putative orthology (see section: *Using increasingly divergent genomic reference species for RNA-Seq analysis*).

**Figure 5:**
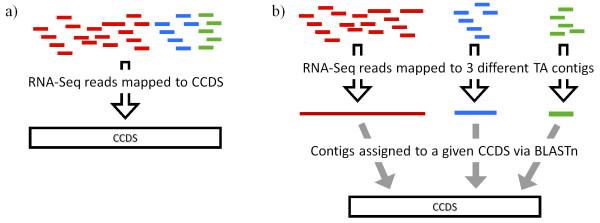
**Diagram of the two methods used to assign RNA-Seq reads to CCDS. a)** RNA-Seq reads are mapped directly to the CCDS dataset, **b)** RNA-Seq reads are mapped to a TA and then the TA contigs assigned to CCDS via BLASTn.

Given the highly fragmented nature of *de novo* TAs, many genes are likely to be represented by several non-overlapping contigs. Using such a TA as a scaffold for mapping RNA-Seq reads will result in the mRNA reads for a given gene being split among these contigs. Thus, in order to create an expression profile at the whole gene level, which is equivalent to mapping RNA-Seq reads to a predicted full length gene model, contigs of the same gene need to be grouped in order to sum their RNA-Seq reads. Comparing the expression profiles produced when using *de novo* assemblies as the mapping reference versus using the *H. sapiens* CCDS dataset was achieved by using CLC Genomics Workbench to map paired-end Illumina to the CCDS unique gene set and to two of the *de novo* TAs. The two TAs investigated in this part of the study were TA_Illprs and TA_Illprs&454, since the former represents the data likely to be acquired from a given RNA-Seq experiment, while the latter represents a higher quality TA within the reach of most non-model research systems. Reads mapped to the TA contigs were then assigned to CCDS genes by assigning each contig of a TA to a single CCDS via BLASTn (Figure 5).

We first assessed the effects of scaffold on technical replicate data using three pairs of Illumina sequencing runs (pair 1: 50nt paired-end reads with an average insert size of 200nt, totalling 16.4 million reads [Genbank: SRA012427: SRR039628/29]; pair 2: 50nt paired-end reads with an average insert size of 200nt, totalling 15.5 million reads, [Genbank: SRA012427-SRR039630/31]; pair 3: 50nt paired-end reads with an average insert size of 200nt, totalling 14.9 million reads, [Genbank: SRA012427-SRR039632/33]). Each of these runs were independently mapped to both the CCDS dataset and to the two TAs (TA_Illprs and TA_Illprs&454). Expression was initially measured as the number of unique reads that map to a whole gene. Scaffold had little effect on technical replicates, with replicates having > 0.9 correlation (Spearman’s rank correlation coefficient, ρ; Additional file 3: Figure S 2). These correlations were further investigated using a MVA (minus versus average) plot [22], which provides insights into abundance-dependent biases [23-25]. For this analysis expression was measured using RPKM (Reads Per Kilobase of exon model per Million mapped reads) values [26]. As is typical for RNA-Seq technical replicates, agreement among replicates is highly dependent upon expression level, with low expressed genes showing less agreement among replicates [25] and this effect was essentially identical across scaffolds (Additional file 4: Figure S 3).

**Figure 6:**
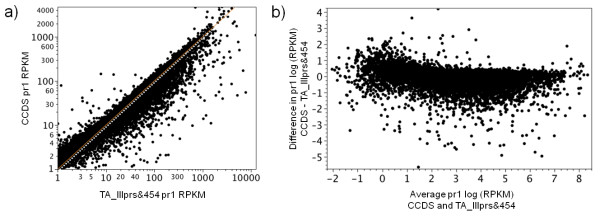
**Comparison of the RNA-Seq expression levels produced from different mapping methods. a)** Y axis shows the RPKM values when reads are mapped directly to the CCDS, X-axis shows the RPKM values when mapping the same CCDS genes via the TA_Illprs&454 scaffold. Method for mapping via TAs is showing in Figure 5b. **b)** Y axis shows the number of differences between RNA-Seq reads mapped (in RPKM) either directly to the CCDS or via the TA_Illprs&454 scaffold [log(CCDS) – log(TA_Illprs&454 scaffold)]. X-axis shows the average of the log RPKM for CCDS genes using the two mapping methods.

We then compared the mapping of one set (pair 1) of the RNA-Seq read data directly to the CCDS dataset versus two of the TAs to assess the performance of *de novo* TAs for whole gene expression analysis. Directly mapping to the CCDS dataset recovered expression data for 15,763 genes, while going through TA_Illprs or TA_Illprs&454 provided data for 10,761 and 12,860 genes respectively. However, of those genes (CCDS) that did have expression data in terms of number of unique reads from both methods the correspondence between them was extremely high (CCDS vs. TA_Illprs Spearman’s ρ = 0.94, P < 0.0001; CCDS vs. TA_Illprs&454 Spearman’s ρ = 0.95, P < 0.0001; Additional file 5: Figure S 4). Expression as RPKM was also measured for one comparison – that of mapping to CCDS vs. to TA_Illprs&454 (Figure 6a), the results of which also showed a high degree of correlation. A MVA plot was used to further assess the relationship between the CCDS and TA_Illprs&454 mapping (Figure 6b). The distribution of disagreement between the two mapping methods was not a function of expression since the TA_Illprs&454 mapping had a higher level of expression than the CCDS mapping across the range of expression values. Two separate and technical causes were observed. First, calculation of the RPKM values for the CCDS mapping used the length of the CCDS gene, while for the TA_Illprs&454 mapping, only the length of the CCDS gene covered by the TA was used (in order to reflect the *de novo* aspect of the mapping). This caused an inflation of the TA values. Second, the two mapping approaches were different. The direct mapping only quantified the reads that mapped uniquely to a given CCDS. Although the TA mapping used the same approach for mapping RNA-Seq reads to the individual contigs of the TA, each contig was assigned to its best CCDS BLAST hit. This approach allowed for the collection of 5’ and 3’ UTR regions into contigs that also overlapped with CCDS genes, significantly inflating the TA mapping reads.

### Using increasingly divergent genomic reference species for RNA-Seq analysis

For species lacking a well assembled genome, annotating RNA-Seq reads is problematic. One route is to map reads to a *de novo* TA and then assign each TA contig (and therefore the associated reads) to a unique gene through a BLAST search against the reference gene set of the nearest Genomic Reference Species (GRS). However, the evolutionary divergence between the target species (the species of interest) and the GRS is likely to be a significant source of bias and error. For genes with high rates of evolutionary change there is a decreased likelihood of successful homology matching between the TA contigs of the target species and GRS gene. It is therefore expected that as evolutionary distance increases, the ability to group contigs to a particular gene will decrease and potentially be accompanied by an increase in incorrect assignment of contigs to putatively orthologous genes. In addition, with increasing evolutionary distance, the most accurate expression information is likely to come from a biased set of genes, notably those having a high level of expression and low rates of evolutionary change (e.g. housekeeping genes). Here we explore the magnitude of this effect by studying the decrease of information content and accuracy of RNA-Seq data gene assignment with the use of increasingly divergent GRS as proxy references.

**Figure 7:**
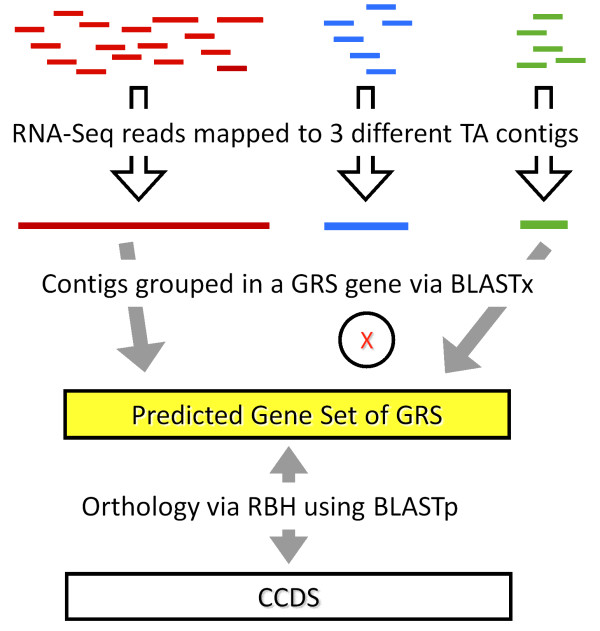
**Overview of the non-model species RNA-Seq mapping strategy for inferring ‘gene’ grouping of RNA-Seq read data.** Displayed are RNA-Seq reads that are mapped to three different TA contigs. The red and green contigs (DNA) are assigned to the same gene of the GRS (protein) via BlastX. However, due to divergence between the target species and the genomic reference species (GRS), the blue contig is not, resulting in only the red and green RNA-Seq reads being assigned to this GRS ortholog. In order to compare the expression data inferred from these GRS groupings to that obtained by directly mapping RNA-Seq reads to the CCDS genes, the orthology between GRS and CCDS genes was determined using the Reciprocal Best Hit (RBH) via BLASTp. This method can be compared to the method outlined in Figure 5b.

Six species of increasing divergence from *H. sapiens* were chosen as genomic reference proxies, spanning a range of 5 to 160 million years divergence. This range was chosen as a likely range researchers may encounter in their choice of a GRS. RNA-Seq reads were mapped to the most cost effective *de novo* TA that also performed well (TA_Illprs&454) and each contig from this assembly was assigned to the predicted genes from each divergent species using BLASTx (with GRS genes as protein sequences). The orthology relationships were also determined between the *H. sapiens* CCDS and GRS gene sets using the Reciprocal Best Hit method [27-29] via BLASTp (see Figure 7 for a diagrammatic overview). The results of using these GRS proxies were then compared with directly using the predicted gene set of the target species (the *H. sapiens* CCDS dataset).

#### Transcriptome coverage when using proxy GRS

**Figure 8:**
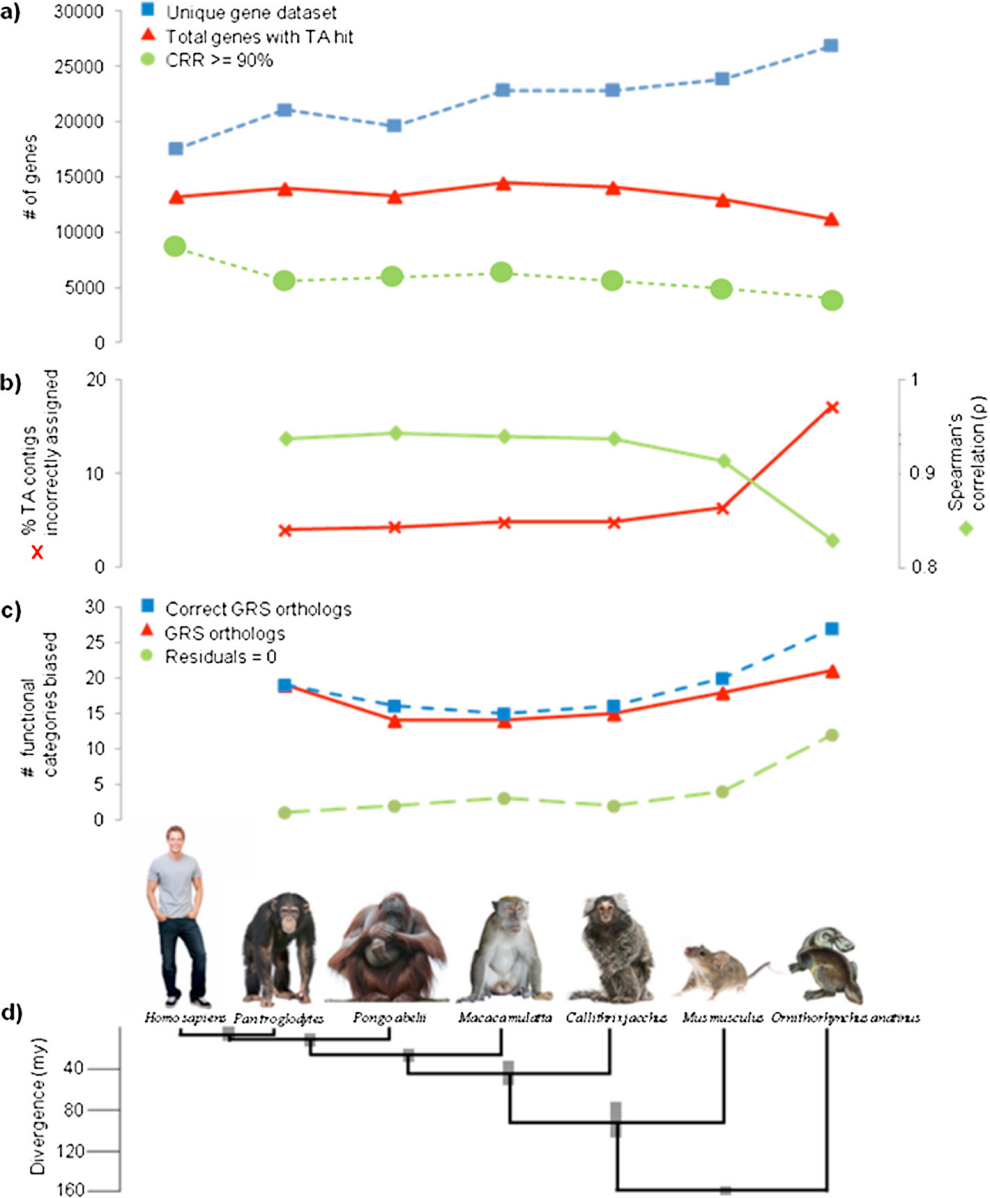
**Assessment of error and bias using increasingly divergent genomic reference species as proxies. a)** Number of genes in different datasets derived using either *H. sapiens*, or each of the six species evolutionarily divergent from *H. sapiens* as the genomic reference. Lines are as follows: Blue square - the total number of genes in the filtered species dataset; Red triangle - the number of genes that have TA contig hits; Green circle - Number of genes with CRR > = 90%; **b)** Blue diamond - Comparison of the Spearman’s correlation (ρ) for expression values obtained through annotating TA contigs using the CCDS dataset and using the proxy GRS datasets; Red squares - Level of error incurred through using divergent GRS to annotate TA measured as the percentage of TA contigs incorrectly assigned to CCDS; **c)** Bias obtained through using GRS as proxy datasets (number of GO and/or KEGG categories): Red triangles - GRS genes orthologous to human CCDS genes; Blue squares - subset of the GRS orthologs that have only TA contigs that are correctly assigned to them; Green circles - residuals from a graph of expression values obtained via mapping to the TA and then annotated either directly to the CCDS or to a GRS gene set. Significance is at p < 0.05 in all cases; **d)** Approximate divergence times of proxy GRS from *H. sapiens* (taken from [30-33].

We first assessed the utility of the GRS grouping approach by calculating the number of GRS genes that hit the *de novo* TA using BLASTx (Figure 8a). There is no appreciable difference in the number of genes of Chimpanzee, Orangutan, Macaque and Marmoset and that of the human dataset (CCDS) that have TA contigs assigned to them. However, there is a decrease in the number of genes that have a TA contig hit when using the Mouse gene set and a further drop when using Platypus. There is thus a decrease in the number of genes that can be identified in the *de novo* transcriptome with evolutionary divergence, however this effect is only observed at the high end of divergence.

We also expect the number of orthologous relationships between TA contigs and a particular GRS gene i.e. gene coverage, to decrease with increasing divergence. To measure this effect we determined the CRR value for each of the GRS genes (or CCDS in the case of *H. sapiens*) (Figure 8a). In this case there is an immediate effect of using a proxy reference genome, with the number of genes having a CRR > =90% decreasing from that of Human (7353 genes) to that of the next closet relative, the Chimpanzee (5614 genes), which is a drop of 24%. This decrease then levels out, with only a 4.3% decrease across *ca.*100my to Mouse (CRR > =90% in 5370 genes) and finally reaches its lowest level in Platypus, which only had 4645 genes with a CRR > =90%. These results suggest that the loss of RNA-Seq read information when using a proxy GRS derives from one or more contigs not being assigned to their GRS gene due to lack of orthology. This will lead to a loss of sequence and associated expression value per gene. However, this loss does not increase in a linear fashion with evolutionary distance from the target species.

#### Effect of using proxy GRS on expression signal: introduction of error

In order to determine how much expression signal is lost via reduced contig assignment when using divergent species as proxy references, expression values were calculated for GRS genes that are both represented in the *de novo* TA and have an RBH ortholog in the CCDS dataset. By grouping TA contigs that hit GRS genes orthologous to CCDS, we could compare the expression signal between mapping to a CCDS via a TA (Figure 5b) and to the GRS dataset via a TA (Figure 7). The correlation is fairly high for all taxa (Chimpanzee, Orangutan, Macaque, Marmoset: Spearman’s ρ = 0.94; p < 0.0001; Mouse: Spearman’s ρ = 0.91; p < 0.0001) except Platypus (Spearman’s ρ = 0.83; p < 0.0001). Thus, while there was some loss of expression signal (expected as fewer contigs are assigned to GRS datasets compared to the CCDS), using the GRS as’gene’ grouping proxies was still informative.

**Figure 9:**
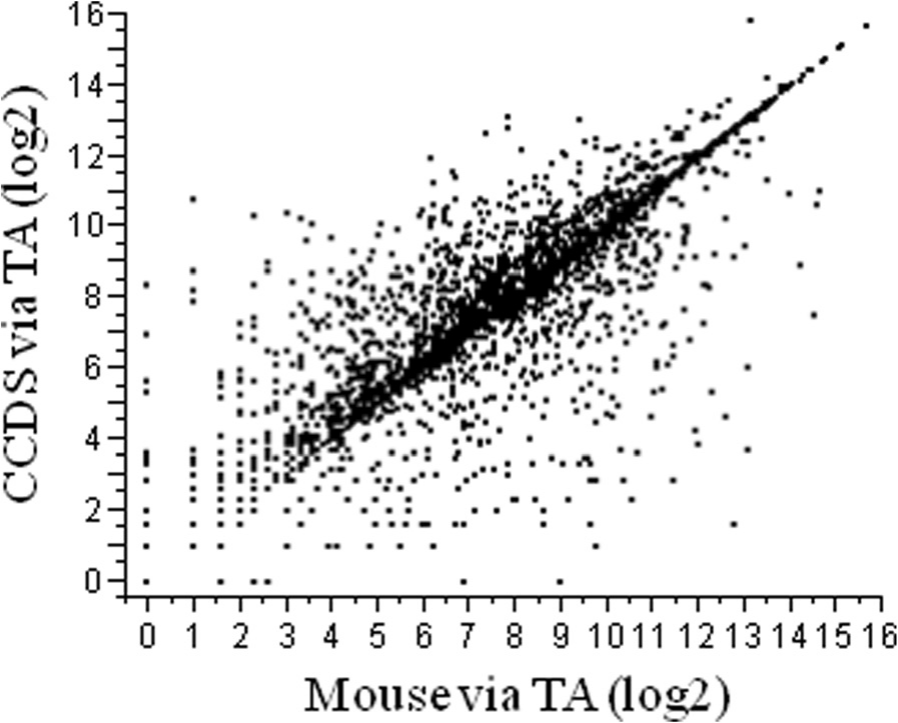
**Quantiative gene expression results comparing results from direct mapping vs. using a Mouse proxy.** Comparison of expression levels (log2) between genes identified via BLASTn of TA contigs and the *H. sapiens* CCDS dataset (Y-axis), and genes identified first via BLASTx of TA contigs and the Mouse dataset, and then BLASTp RBH of Mouse dataset and the Human dataset (X-axis). Each point represents a CCDS gene. Points above line of unity include genes that lose contigs through no hit in the Mouse dataset.

In order to explore sources of error when using increasingly divergent GRS proxies, we further examined the differences in expression values obtained via mapping TA contigs to the CCDS or via one of the GRS (Figure 9). Points above the line of unity would be CCDS that have fewer contigs assigned to it through utilisation of GRS to annotate the TA. Points below the line of unity, as well as some above it, are CCDS that have contigs wrongly assigned to them during the BLAST search, resulting in a greater number of reads than the direct CCDS mapping method. The extent of this error can be calculated by identifying the true subset of GRS genes that are orthologous to Human genes in the CCDS dataset. Since we previously assigned, via BLASTn, TA contigs to CCDS genes, and have established CCDS to GRS RBH orthologs with high confidence using BLASTp, whether a given TA contig assignation to GRS gene was correct can be determined (correct when TA contigs are assigned to the same CCDS via direct BLASTn or via BLASTx to a GRS gene and which in turn matches the same CCDS via BLASTp). The BLASTp RBH between the GRS and CCDS datasets was assumed to be robust and so if a contig was wrongly assigned to a CCDS (via the GRS dataset), it is likely that BLASTx is the source of error.

The level of error (percentage of TA contigs wrongly assigned to a CCDS via GRS dataset) was found to increase as evolutionary distance from Human increases (Figure 8b). The error for Chimpanzee was fairly low, at 3.96%, and similar to Orangutan (4.20%), Macaque (4.80%) and Marmoset (4.80%). Utilisation of Mouse resulted in 6.30% of the contigs being wrongly assigned, while for Platypus error increased massively to 17.06%. As mentioned above, error is likely to occur during the BLASTx of TA contigs and GRS genes (proteins). We investigated whether increasing the stringency of the BLAST parameters decreased the level of error for two of the GRS: Chimpanzee and Mouse. In both cases it did, with Chimpanzee now having 2.49% error, and Mouse 4.43% error. As expected this decrease was highest for the more divergent Mouse. A repercussion of increasing the threshold identity in the BLAST searches is that fewer genes were annotated in the *de novo* TA (Chimpanzee: 12922 (decrease of 7.28%); Mouse: 11533 (decrease of 11.11%). The BLASTx parameters used must therefore be a tradeoff between the size of the transcriptome that can be annotated by using a divergent GRS, and the level of error accepted.

#### Ascertaining the level of bias when using proxy GRS

We further investigated the potential sources of error and bias by gene set enrichment analysis using the GO and KEGG functional categories of the Human CCDS genes (Figure 8c; Additional file 6: Table S 2, Additional file 7: Table S 3, Additional file 8: Table S 4). First, we assessed whether there was any bias in the GRS genes identified as CCDS orthologs by TA hits of the GRS datasets. For all the taxa investigated we observed a significant bias in many functional categories although there is no significant pattern of an increase in this bias with evolutionary divergence. However, when only genes that had no contigs incorrectly assigned to them were analysed a different picture emerged (Figure 8c). First, there was an increase in the number of biased functional categories for all comparisons, although this was modest for most taxa. Second, there was an increase in bias with divergence that mirrored the pattern of error. This trend was mainly due to the highly divergent Platypus, where the number of biased functional categories rose from 21 to 27 when error was removed. This pattern suggested that the increased error in the most divergent species masked the higher bias in those species.

Next we investigated bias in the gene expression results from the two different mapping approaches. We took the residuals from a fit of expression levels from the direct mapping of TA (TA_Illprs&454) to CCDS against the expression levels obtained using GRS proxies to annotate the TA contigs. Residuals had a value of 9 when genes had identical expression levels in both methods. Residuals were positive when they had fewer reads mapped to them from the method using GRS proxies to annotate the TA contigs, and residuals were negative when CCDS genes had more reads mapped to them using the GRS proxies to annotate the TA contigs method. We expected that highly expressed and evolutionarily conserved genes would be over-represented among those having a residual of 0, and that this bias would increase over evolutionary time. We therefore assessed whether there was any unequal distribution of gene functional categories among those having residuals = 0 vs. those having residuals ≠ 0. We found that there is a significant bias in a low number of functional categories for all taxa, except in Platypus where the number of biased functional categories increases threefold above Mouse (Figure 8c). When investigating whether there was a difference between positive and negative residuals, we found no biased categories in any taxa (results not shown).

Our final analysis looked at general trends in the types of functional categories that were over and under-represented in our analyses (Additional file 6: Table S 2, Additional file 7: Table S 3, Additional file 8: Table S 4). Mitochondrion (GO:0005739) and protein binding (GO:0005515) categories were nearly always over-represented when using GRS to annotate TA contigs, and in comparisons with Platypus, transcription and translation related categories (e.g. translation (GO:0006412), RNA splicing (GO:0008380), ribosome (GO:0005840)) were also over-represented. Typically under-represented categories included a diversity of biological functions, from sensory perception of smell (GO:0007608) and defence response to bacterium (GO:0042742), to signal transduction (GO:0007165). In general then, it appears that genes in the over-represented category, primarily represented by comparisons with Platypus, included genes with very conserved housekeeping functions, while those being under-represented included categories of genes known to undergo more complex evolutionary dynamics (e.g. birth-death dynamics).

## Discussion

Analysing RNA-Seq data for gene expression has traditionally required genomic resources for the species of interest in order to map and annotate reads. Greater sequencing depth and read length, more advanced assembly software [6,34] and most importantly, lower costs, now make RNA-Seq an attractive alternative to designing and using custom microarrays for researchers wanting to study the transcriptomes of species that don’t have genomic resources. For such non-model species one route to using RNA-Seq for expression insights is to perform *de novo* transcriptome assembly and use this assembly as a scaffold for quantitative RNA-Seq mapping. While this has been done using the 454 platform (e.g. [9]), the small number of reads typically provided per run (ca. 1 x 10^6^) makes this perhaps only accurate for the most highly expressed genes. Currently in the literature there is much discussion about how many RNA-Seq reads are necessary to generate repeatable quantitative measures for middle to low expressed genes, with emerging empirical results suggesting at least 10 to 30 million reads are necessary ([25,35], but see [36]). This strongly suggests that using the Illumina platform, which can provide two orders of magnitude more reads for less than half the cost of the current 454 technology, is the best way forward for quantitative expression analysis. Thus, here we have assessed the performance of Illumina sequencing data in the non-model species context.

To date, only a handful of studies have applied the Illumina approach for quantitative RNA-Seq expression analysis in non-model species. In their investigation of the transcriptome profiles of parasitized vs. non-parasitized *Plutella xylostella* moths, Etibari and colleagues assembled all of their Illumina reads into a *de novo* transcriptome and consequently used this as a scaffold for mapping their differently parasitized groups. Annotation of the *P. xylostella* transcriptome used a BLAST search in NCBI [18], allowing them to identify differential expression of many metabolic and immune genes. A different study developed a pipeline that facilitates the assembly and annotation of non-model species transcriptomes through utilisation of the genomic resources of related organisms. This method allowed the researchers to perform expression profiling and also to increase the quantity and quality of sequence data available for their targeted species, the Chinese hamster [17].

The current study was motivated by questioning the general accuracy of the *de novo* approach exemplified by these two studies. While their conclusions are well justified, here we have worked to attain a deeper understanding of the potential errors and biases that might underlie such analyses. One major concern is the level of bias in the genes that are finally included in the analysis. Genes assembled and annotated are not likely to be a random sample of the genome, since highly expressed genes will likely be assembled and annotated best. Etibari et al. [18] found no bias in GO terms between their *de novo* transcriptome assembled for *P. xylostella* compared the silkworm *Bombyx mori*, which diverged approximately 120 million years ago (Wheat & Wahlberg, in review). However, the GO terms that are liable to be shared between these two Lepidoptera are themselves likely to be highly biased, as the greatest number of *B. mori* genes having annotations are those in common with the genomic reference species *Drosophila melanogaster*. Given the deep divergence of *D. melanogaster* from *B. mori*, which last shared an ancestor approximately 330 million years ago (Wheat & Wahlberg, in review), the only genes likely to be functionally annotated are those with highly conserved function and constrained evolutionary dynamics. Such housekeeping genes are thus very likely to be those highly expressed in *P. xylostella*, and thus unbiased with respect to the annotated genes in *B. mori*. The Birzele et al. [17] study used Velvet to assemble their transcriptome. This is of concern given recent comparative assessment of transcriptome assembly software packages, which found Velvet to perform among the worst software programs for use upon their transcriptome dataset [37]. We therefore wished to use an assembler which had previously been shown to perform well [37] and so chose the CLC method.

In order to assess the performance of RNA-Seq, we addressed several steps of this approach, beginning with *de novo* transcriptome assembly. The effect of sequencing technology and volume of data upon the quality of the *de novo* TA was assessed by comparing 8 different TAs using RNA-Seq data for *H. sapiens*. In comparison to previous examinations of assembly performance that have used simulated data [37,38], using data from *H. sapiens* gave us a unique combination of genomic insights and real world data. We find, as have others, that the standard metrics commonly applied may lead to a sub-optimal choice of TA [37]. While metrics solely based on contig lengths suggested that TAs composed of only Illumina reads performed best, in-depth investigation using genomic resources showed a different picture. By using BLAST to identify putative orthologous relationships between TA contigs and the predicted gene set of humans, the aligned region between the two could be determined. By dividing this aligned length by the full length of the predicted gene provides a ratio indicating how much of the coding region a TA contig has successfully reconstructed. Here we have used this approach to calculate the entire amount of the predicted gene covered by all the different TA contigs in a given assembly, and referred to this as the contig reference ratio (CRR). After such comparison between TA contigs and their putative ortholog appeared in the first *de novo* transcriptome assembly [12], similar ratios have been developed (e.g. [39]). We find the three most informative ratios are for: 1) all possible TA contigs (all CRR), 2) the longest TA contig per ortholog (longest CRR), and 3) the sum of the ortholog length covered by all the TA contigs, which is then divided by the full length of the ortholog (sum CRR). While informative, all CRR, which was used by O’Neil et al. 2010 [39], inflates assumed TA performance since several contigs for the same gene may be quantified while providing no information as to the total amount of each ortholog a given TA covers. Longest CRR is perhaps the single best metric for assessing TA performance. Ideally, the best TA would predict single contigs that covered the full length of each transcript, as well as the different isoforms, without any over prediction. Here we have used the longest CRR only once, for assessing our TAs (Additional file 2: Figure S 1) and this provided very similar insights to that of our sum CRR results. Throughout our paper we have almost entirely used a sum CRR because we wish to know how much of each gene we have covered in our TA, since maximizing coverage is necessary in order to generate a good scaffold for mapping the RNA-Seq data and this information is combined on a per gene basis.

Availability of the CCDS predicted gene set allowed us to ascertain the level of TA coverage for each gene. Although pure Illumina-based TAs had fewer and longer contigs than both the pure 454 TA and the hybrid TAs (composed of both Illumina and 454 reads), the pure Illumina TAs also had a much lower coverage of the transcriptome compared to the hybrid assemblies, at both the individual gene and total transcriptome level.

Using the hybrid TA as a scaffold produced RNA-Seq mapping results that were similar to directly mapping to the CCDS predicted gene set from the *H. sapiens* genome. Although there were approximately 2000 fewer genes mapped when using the TA as compared to the CCDS gene set, the correlation in whole gene expression values between these two methods was extremely high (Spearman’s ρ = 0.94). Similar high levels of correlation were observed across technical replicates when mapped to the hybrid TA assembly. While additional improvements could be made in the *de novo* approach, the correlation between the two approaches is already much higher than that observed in comparisons between RNA-Seq and microarray results (Spearman’s ρ = 0.73; [40]). Thus, hybrid TA assemblies, combining Illumina and 454 reads, emerge as the best assemblies and scaffolds for RNA-Seq mapping. We should note that this study has utilised Illumina RNA-Seq data that was available at the time, technology is advancing at a rapid rate and the quality of *de novo* transcriptomes that can be assembled with the latest sequencing data (e.g. Illumina’s HiSeq 2000: http://www.illumina.com/systems/hiseq_2000.ilmn) will likely surpass what we show here. Thus this study should be taken as a comparative study, and a conservative guide.

After mapping RNA-Seq reads to a TA (whether it be a *de novo* assembled one, or a transcriptome already available for the species), the contigs need to be assigned to genes for grouping and functional annotation. In non-model species, the ability to obtain significant biological insights into gene expression variation is limited by gene functional annotation. In model systems obtaining gene expression values and assigning these to a growing number of genes of likely biological function is well developed. Non-model systems necessarily must tap into this reservoir of data using BLAST and assumptions of gene function homology, and the genome or gene set of a related but potentially very divergent species. While this approach can be successful (e.g. [12,37]), what effect does increasing evolutionary distance from the focal species have upon functional insights?

Gibbons and colleagues investigated the accuracy of ortholog prediction between increasingly divergent species, using RNA-Seq data from one species and genomic proteome data from a GRS [41]. They observed a decrease in the number of successfully identified orthologs (contig/GRS gene pairs) with increasing divergence. Their study spanned a time-frame of 300 million years from the target species, with the two youngest GRS being 40 and 150 million years divergent from the focal species. Here we sought to investigate whether a negative effect of divergence is observed within the 0–150 million year time frame. Although we expected that as evolutionary distance increased between the GRS and *H. sapiens* there would be a significant decrease in the number of genes in the GRS gene sets that found a hit in the *de novo* TA, this effect was weak up to 100 million years of divergence (Figure 8a). A similar effect was observed when assessing the amount of each gene that was covered by TA contigs. Thus there is little negative effect of using GRS as reference datasets for the grouping contigs as divergence increases up to 100 million years, although beyond this age, the number of genes having good coverage assigned decreases.

These results also suggest that the use of proxy GRS up to 100 million years divergent from *H. sapiens* for grouping TA contigs might result in only a small bias on expression levels compared to directly mapping RNA-Seq reads to the CCDS dataset. Within this time frame proxy GRS are also likely to enable successful measurement of expression levels as a high correlation in expression between these two methods was found in all cases, even when comparing *Mus musculus* (mouse), which is c. 75–91 million years divergent from *H. sapiens* (Figure 8d; [30-32]. However, expression results using these divergent species as proxy references also suffered from a level of incorrect assignment of TA contigs to genes, and this negative effect was found to increase with evolutionary distance (Figure 8c). While this effect was moderate using GRS species up to 100 million years divergent, comparisons using Platypus as the GRS showed both a dramatic increase in incorrectly assigned TA contigs and a lower correlation with the CCDS mapped expression values (Figure 9b). This identified error was mirrored in the gene set enrichment analyses, as incorrect contig assignment should be greatest for genes that have higher evolutionary rates, or conversely, lower for constrained genes (Figure 8c). Error is likely to accrue during the BLASTx search of the TA contigs against the GRS dataset, and indeed when this BLASTx was repeated for two of the GRS using more stringent parameters of identity less error was encountered. However, a repercussion of this was reduced coverage of the transcriptome in terms of genes that could be annotated.

Several important limitations of our approach should be noted. First, there are certainly many species that do not have a genomic reference species less than 100my. While our approach would certainly aide such projects, researchers should be aware of the error and bias inherent in such analyses. Fortunately, as the genomics era progresses available genomic reference species will increase. Second, a large class of genes will lack homology between species, and this will increase with divergence. Such orphan genes are likely to be involved in species-specific adaptations and potentially the most ecologically and evolutionary interesting aspects of a species transcriptome [see [42] for a review]. Therefore, there is a high likelihood that insightful results reside in careful analysis of that part of the transcriptome that does not hit a proxy reference genome and for which no known biological function is established. Our analyses suggest that *de novo* analysis of orphan genes will be most insightful when such genes are assembled to their full length. Careful examination of TA contigs for long open reading frames flanked by both 5’ and 3’ UTRs may prove useful for such assessment (e.g. looking for the polyadenylation signal). Overcoming the bias against studying orphan genes is a challenge facing the entire research community.

A third limitation arises due to variation in recent gene duplication events among individuals, commonly referred to as Copy Number Variants (CNV). When young, CNVs can be very difficult to detect in RNA-Seq data. When mapping RNA-Seq reads back to a full genome, which is usually derived from a single individual, differences among individuals in their CNV with reference to the genome can result in reads from several independent loci being mapped to a single locus, resulting in a spuriously inflated measure of single locus expression. This is certainly the case in the *de novo* approach we use here to obtain whole gene expression levels, as the contigs we assign to the same gene may derive from incomplete transcript assembly as well as recent duplication events. Expression differences detected between biological groups using this whole gene approach necessarily must be studied in more detail, to assess the causal basis of the signal. We argue that this is true in both model and non-model systems alike, where there are likely to be significant differences between the scaffold genome and the individuals having their RNA sequenced. Finally, this is similarly true for splicing isoforms, as our whole gene approach pulls together expression across exons for the entire gene. To the extent that differences among groups arise from expression differences solely in specific exons, this will give rise to expression differences that necessarily must be investigated further to determine whether this difference is evenly distributed across the entire gene. A final point of importance is regarding the choice of transcriptome assembler. Many papers are still emerging where groups have used poorly performing assembly software to assemble their transcriptome data. Our results might not be obtainable with such software, especially as few programs handle hybrid data well. Thus we encourage researchers to be aware of the latest advances in transcriptome assembly and use methods shown to perform well with their generated data [37]. In sum, our whole gene expression quantification provides a robust starting place for the identification of gene expression differences whose biological basis will require more detailed study, as should be common in any RNA-Seq study regardless of genomic resources.

## Conclusions

Our findings indicate that RNA-Seq data from non-model species can be successfully *de novo* assembled into quality transcriptomes. These assemblies can then be used with high performance, as scaffolds for mapping RNA-Seq read data for quantitative whole gene expression analyses. In order to functionally annotate *de novo* transcriptomes, proxy genomic reference species up to approximately 100 million years divergence from the target species can be utilised, generating results similar to those produced from using high quality predicted gene sets as scaffolds. Although there is a reduction in the size of the annotated portion of the transcriptome assembly when using proxy reference species, and there is a significant amount of error, these effect sizes are relatively small until past 100my divergence. The use of more stringent parameters in BLAST searches reduces this error, but this also decreases the number of genes that are able to be annotated, thus producing a trade-off researchers should be aware of. The level of bias in the genes that are able to be annotated in the resultant transcriptome is also an important consideration, as highly divergent (and often the most interesting) genes are potentially missing from the analyses. As sequencing technology advances, as it will already have done since this study, the quality and amount of RNA-Seq data that will be produced and the ability of researchers of all disciplines to assemble and annotate transcriptomes of non-model species will increase dramatically, making all species amenable to such studies in the future.

## Methods

### RNA-Seq data

In order to assess the potential of *de novo* RNA-Seq analysis for non-model species, datasets from both Illumina (http://www.illumina.com) and 454 GS FLX (http://www.454.com) were needed from a species having a wealth of genomic information. Searches of available databases revealed that sufficient data was available for *Homo sapiens*. RNA-Seq data for all analyses were generated from the same RNA reference sample (Human Brain Reference RNA) of the MicroArray Quality Control (MAQC) project (MAQC Consortium 2006; [43]). Data from two Illumina ([Genbank: SRA012427: SRX018974-79], 3 sets of paired-end Illumina runs, one run per lane; [Genbank: SRA010153: SRX016366] - one full plate of Illumina, 7 lanes) and one 454 GS FLX ([Genbank: SRA003647: SRX002933 & SRX002935] - 11 runs, each one a half plate) experiments were downloaded from the Sequence Read Archive (SRA) at NCBI (http://www.ncbi.nlm.nih.gov). The data were imported into the CLC Genomics Workbench 4.7 (http://www.clcbio.com) and the reads quality and adaptor trimmed from fastq data (where appropriate) using the default settings (Ambiguous limit = 2, quality limit = 0.05). See Additional file 1: Table S1 for the size of the RNA-Seq datasets and the number of reads incorporated into a *de novo* transcriptome assembly.

### Predicted gene sets

A predicted gene set for *Homo sapiens* was downloaded from the consensus coding sequence (CCDS) database [44] at NCBI, build 37.1 (http://www.ncbi.nlm.nih.gov/CCDS/CcdsBrowse.cgi). This gene set was filtered using custom python scripts to identify and remove alternative splice variants and recent gene duplications by self BLAST. For each CCDS that found a hit using BLASTn against another CCDS with > 95% DNA identify for at least 100 nucleotides and an e-value < = 1x10-6, the shorter of the two, or its exact duplicate, was removed. The resulting filtered dataset is hereafter referred to as the CCDS dataset.

Predicted gene sets of 5 species of increasing evolutionarily divergence from *H. sapiens* were downloaded from Ensembl, release 63 (http://www.ensembl.org). These were self BLAST filtered as above, but at the protein level (BLASTp), with removal of the shorter sequence when amino acid identity > 90% over 33 amino acids with an eval < = 1x10^-6^. The species used and approximate divergence times from *H. sapiens* are Chimpanzee (*Pan troglodytes*, ~5-10my), Orangutan (*Pongo abelii*, ~13-18my), Macaque (*Macaca mulatta*, ~20-35my), Marmoset (*Callithrix jacchus*, ~33-50my), Mouse (*Mus musculus*, ~75-91my) and Platypus (*Ornithorhynchus anatinus*, ~160-162my) [30-33].

### de novo *assembly and mapping of RNA-seq reads*

RNA-Seq data were *de novo* assembled into transcriptome assemblies in various combinations using CLC Genomics Workbench. General parameters for assembly were as follows: mismatch cost set at one and both insertion and deletion cost set at two. Other parameters used in the transcriptome assemblies were dependent upon the sequencing platform of the data used. For paired-end Illumina data the minimum and maximum distances between the pairs was 150nt and 250nt. For 454 data, which were unpaired, the assembly parameters also included a length fraction of 0.4 and a similarity limit of 0.85. The minimum contig size for all assemblies was 200 nucleotides. Scaffolding was not performed for the assemblies in this study. Assembly statistics are available in Additional file 1: Table S 1.

Paired-end Illumina RNA-Seq data were mapped to each *de novo* TA and also to the human CCDS dataset using the following parameters in CLC Genomics Workbench. The maximum number of mismatches allowed was two; the minimum length and similarity fraction was set at 0.8; and the maximum number of hits per read was 10. Broken pairs were included in the counting scheme and only unique mappings were counted for expression analyses.

### Bioinformatics

To uniquely assign each TA contig to its best hit CCDS, BLASTn was used and the results filtered according to two criteria: > 95% DNA identify for at least 100 nucleotides and an e-value < = 1x10^-6^. Unique assignment of each TA contig to its putative ortholog in the predicted gene set of a given divergent species used BLASTx, at the cut-off levels: bitscore > = 50, e-value < = 1x10^-6^, over a length of 33 amino acids. A second more stringent cut-off level was used in an assessment of the level of error incurred during the BLASTx: bitscore > = 100, e-value < = 1x10^-25^, over a length of 33 amino acids. Orthology assignment between *H. sapiens* and the other mammalian predicted gene sets, all at the amino acid level using BLASTp, were determined using the robust method of Reciprocal Best Hit [27-29] BLAST at the cut-off levels: >60% DNA identify for at least 33 amino acids and an e-value < = 1x10^-5^. Custom scripts were written for running and parsing all BLAST commands and outputs. NCBI’s BLAST version 2.2.25 was used both locally and at the Centre for Scientific Computing (CSC), Finland. Gene enrichment analyses compared the distributions of GO and KEGG categories between selected lists using the FATIGO tool of the online software suite Babelomics (http://babelomics3.bioinfo.cipf.es; [45]). CCDS genes were assigned their Ensembl header and these identities as input for Babelomics, which assigned functional categories based upon them. The lists were quality assessed prior to use by ensuring only one copy of each gene was used. Parameter settings for FATIGO were as follows: GO levels analysis not inclusive (join levels); Direct annotation through ontology levels; Filter terms by number of annotated genes in DB (Your input genes). A two-tailed Fisher exact test was performed for each FATIGO analysis (n = 30). All other statistical analyses were performed using the software package JMP version 8 (SAS, Inc.).

## Competing interests

The authors declare that they have no competing interests.

## Author’s contributions

EAH assembled the transcriptomes and mapped the sequencing reads, and participated in the study design. CWW conceived of the study and wrote python scripts for BLAST and to calculate the CRR. Both authors analysed the data and wrote the manuscript. All authors read and approved the final manuscript.

## Supplementary Material

Additional file 1 **Table listing the RNA-Seq data used in the *de novo *transcriptome assemblies (TA) and the basic assembly metrics for each transcriptome assembly.** Click here for file

Additional file 2 Figure depicting the CRR distribution of the single longest TA contig for each CCDS gene, for the different TAs. Click here for file

Additional file 3 Panels of figures depicting pairwise comparisons of expression data produced when mapping different technical replicates of RNA-Seq Illumina data to varying templates. Click here for file

Additional file 4 Panels of figures depicting pairwise comparisons of expression data produced when mapping different technical replicates of RNA-Seq Illumina data to varying templates. Click here for file

Additional file 5 **Plot comparing the RNA-Seq expression levels produced from either mapping reads directly to the CCDS dataset, or to a *de novo *transcriptome assembly.** Click here for file

Additional file 6 Table listing the functional GO and KEGG categories that are significantly (p < 0.05) over- or under-represented in the total list of GRS orthologs of CCDS genes that have a TA hit, compared to the total list of CCDS genes. Click here for file

Additional file 7 Table listing the functional GO and KEGG categories that are significantly (p < 0.05) over- or under-represented in a list of GRS orthologs of CCDS genes that only have correctly assigned TA hits, compared to the total list of CCDS genes. Click here for file

Additional file 8 Table listing the functional GO and KEGG categories that are significantly (p < 0.05) over- or under-represented in the list of residuals that equal 0 in a plot of expression levels obtained when mapping TA contigs directly to the CCDS gene set versus mapping the same TA contigs to the GRS dataset (and then using the orthologous genes for comparison purposes). Click here for file
