# Supplementary figures and images for: Quantitative RNA-Seq analysis in non-model species: assessing transcriptome assemblies as a scaffold and the utility of evolutionary divergent genomic reference species
Source: BMC Genomics. 2012 Aug 1;13:361. doi: 10.1186/1471-2164-13-361 (PMC3469347; doi:10.1186/1471-2164-13-361)

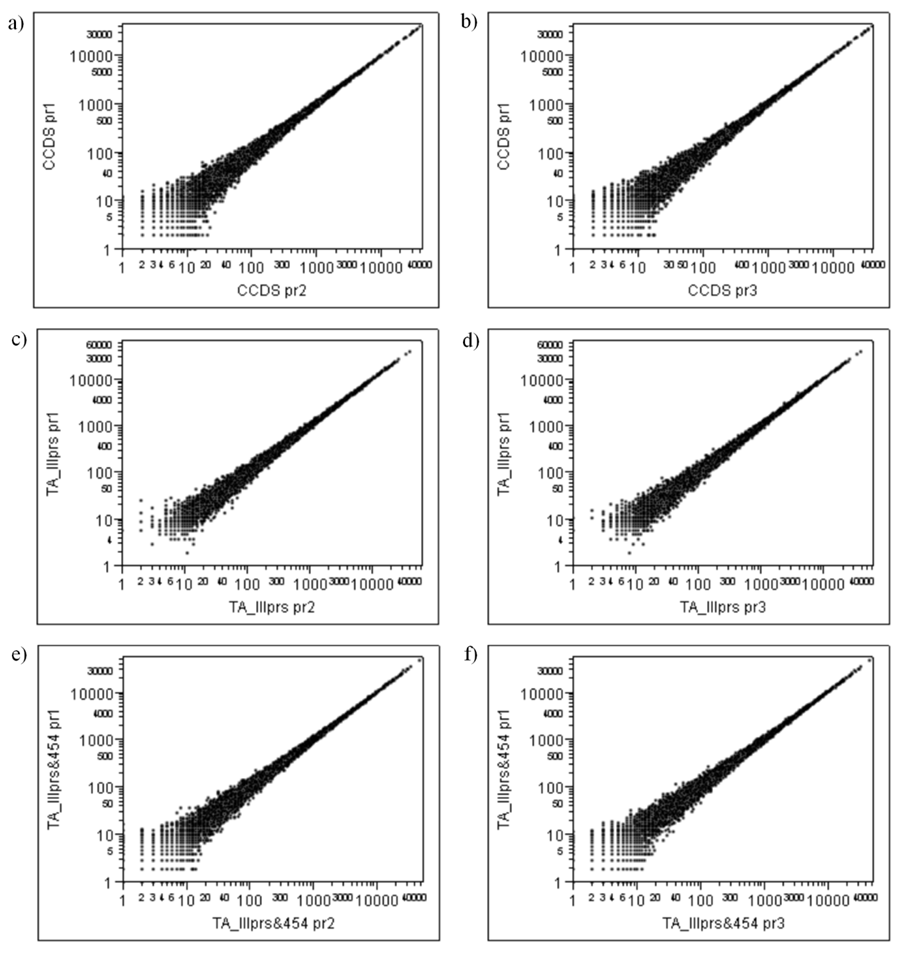

Supplement: Additional file 2 — Figure depicting the CRR distribution of the single longest TA contig for each CCDS gene, for the different TAs. [file 1471-2164-13-361-S2.tiff]

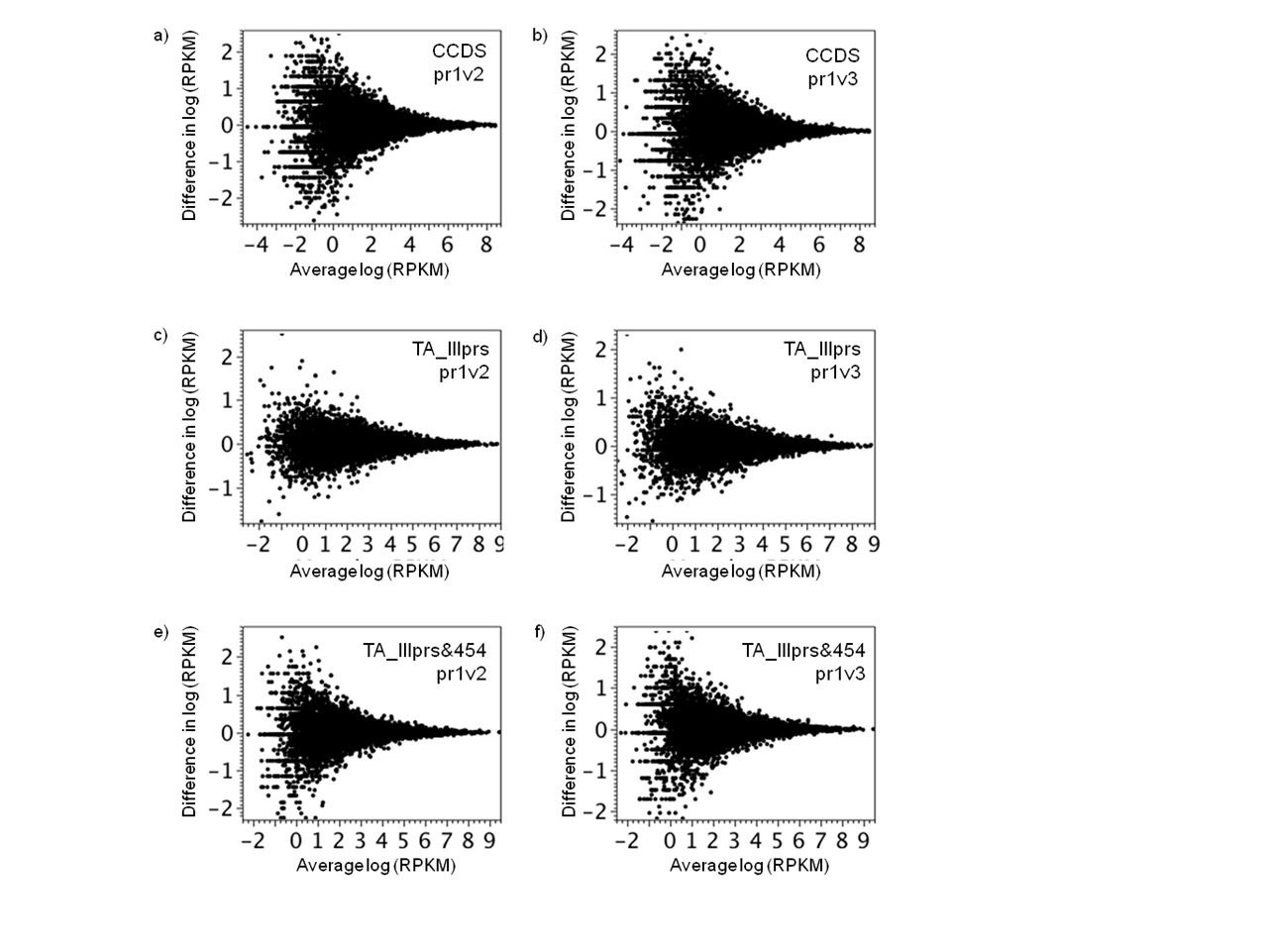

Supplement: Additional file 3 — Panels of figures depicting pairwise comparisons of expression data produced when mapping different technical replicates of RNA-Seq Illumina data to varying templates. [file 1471-2164-13-361-S3.tiff]

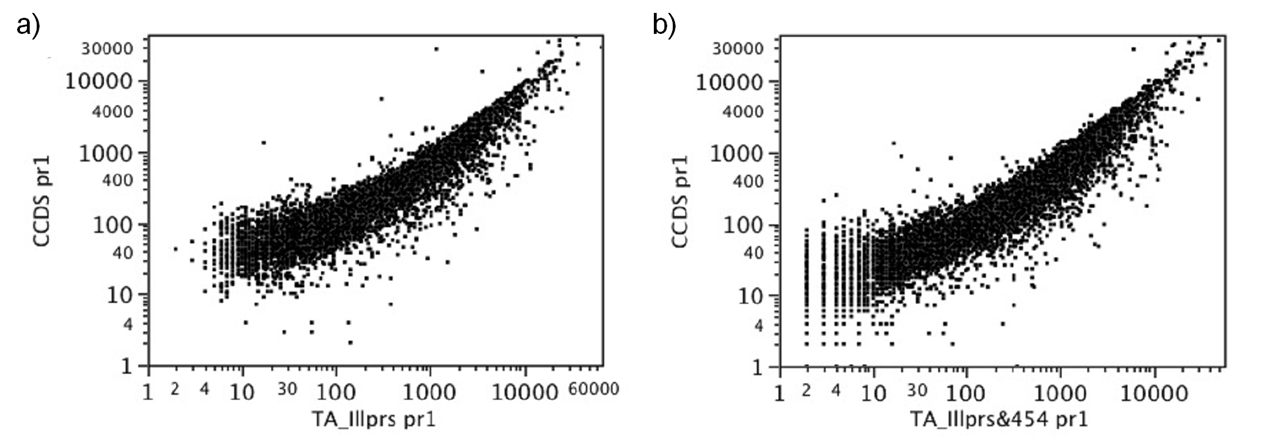

Supplement: Additional file 4 — Panels of figures depicting pairwise comparisons of expression data produced when mapping different technical replicates of RNA-Seq Illumina data to varying templates. [file 1471-2164-13-361-S4.tiff]

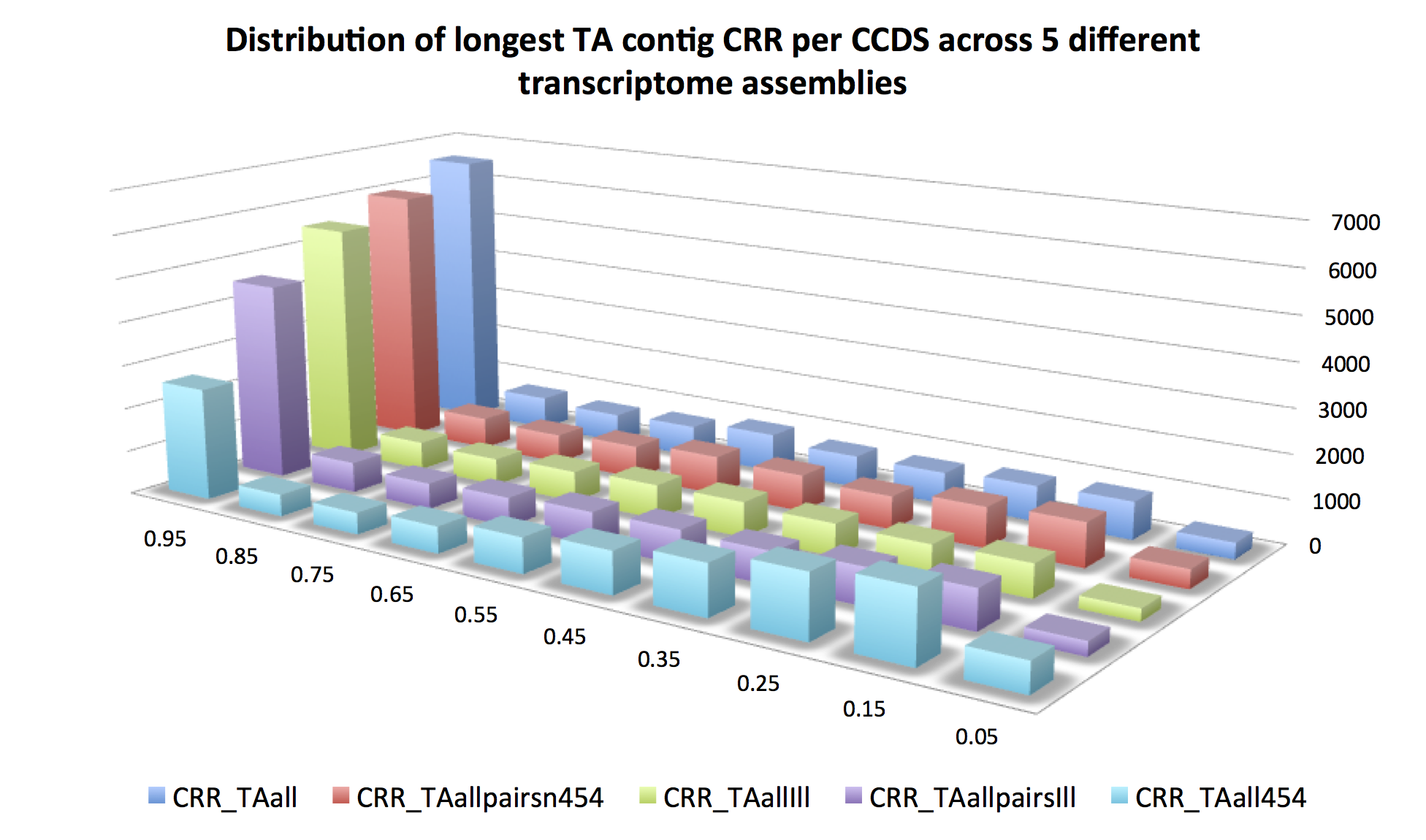

Supplement: Additional file 8 — Table listing the functional GO and KEGG categories that are significantly (p < 0.05) over- or under-represented in the list of residuals that equal 0 in a plot of expression levels obtained when mapping TA contigs directly to the CCDS gene set versus mapping the same TA contigs to the GRS dataset (and then using the orthologous genes for comparison purposes). [file 1471-2164-13-361-S8.tiff]
